# Computed Tomography Angiography for Detection of Middle Meningeal Artery Lesions Associated with Acute Epidural Hematomas

**DOI:** 10.1155/2014/413916

**Published:** 2014-03-13

**Authors:** Wellingson Silva Paiva, Almir Ferreira Andrade, Robson Luis Oliveira De Amorim, Edson Bor-Seng-Shu, Gabriel Gattas, Iuri Santana Neville, Jose Guilherme Caldas, Eberval Gadelha Figueiredo, Manoel Jacobsen Teixeira

**Affiliations:** ^1^Division of Neurological Surgery, University of Sao Paulo Medical School, 255 Eneas Aguiar Street, Office 4080, 05403010 Sao Paulo, SP, Brazil; ^2^Institute of Radiology, University of Sao Paulo Medical School, 470 Alves Guimaraes Street, 05410-000 Sao Paulo, SP, Brazil

## Abstract

*Background.* The natural history of traumatic aneurysms of the middle meningeal artery (MMA) is not well known, but patients with these lesions are more likely to have delayed bleeds. In this paper, we described a series of patients with epidural hematoma who underwent angiotomography (CTA) for MMA vascular lesion diagnosis. * Methods.* Eleven patients admitted to our emergency unit with small acute epidural hematoma were prospectively studied. All patients with temporal acute epidural hematomas underwent CTA and cerebral angiogram at our institution for diagnosis of posttraumatic lesions of middle meningeal artery. The findings of angiotomography and digital angiography were reviewed by radiologist and angiographers, respectively, to ensure that the lesions were readily diagnosed without knowing the results of angiotomography and to compare CTA findings with standard angiogram. * Results.* The causes of head injury were traffic accidents, falls, and aggression. Three of these patients presented traumatic MMA pseudoaneurysm. CT angiography was able to diagnose all of them, with dimensions ranging from 1.5 to 2.8 mm. Conventional angiography confirmed the findings of CT angiography, and the lesions presented with similar dimensions at both methods. * Conclusions.* We believe that angiotomography can be a useful technique for diagnosis of vascular lesion associated with small epidural hematoma.

## 1. Introduction

Acute epidural hematomas (AEDHs) are common traumatic lesions [1]. It is well known that small AEDHs without significant mass effect may be treated conservatively, and their ideal management has not been clearly established thus far [2–4].

Late enlargement of previously small hematomas is a well-recognized clinical occurrence [2, 5]. Patients with cranial fractures crossing over dural arteries or veins are prone to experience rebleeding with consequent hematoma enlargement [2, 6]. In patients who present with fractures crossing the middle meningeal artery (MMA), the possibility of false aneurysm should be kept in mind [2, 7]. Patients with traumatic pseudoaneurysms are more likely to have delayed bleeds, which account for typical prolonged lucid interval [8, 9]. It is important to diagnose and treat these aneurysms at the earliest to prevent catastrophic events. Recent study has suggested that the incidence of posttraumatic pseudoaneurysms is higher than previously thought [2].

Intracranial vascular lesions related to cranial fractures and small AEDHs have not been adequately studied thus far, and their incidence, natural history, clinical relevance, and ideal management have not been well established. Angiograms have been used to identify pseudoaneurysms associated with small epidural hematomas [2]. However, it presents several drawbacks and the associated morbidity cannot be neglected. The role of “less invasive” methods, such as CT angiography, to diagnose these lesions has not been determined thus far. The aim of the current study is to describe the radiological findings in 11 patients who harbor small AEDHs associated with linear cranial fractures crossing over the MMA trajectory and to compare CT angiography (CTA) versus conventional angiography.

## 2. Methods

Eleven consecutive patients admitted to the emergency unit in the Division of Neurological Surgery at University of São Paulo were prospectively studied. All patients with temporal or parietal AEDHs underwent CTA and cerebral angiogram at our institution for the diagnosis of posttraumatic lesions of middle meningeal artery. Eight patients were male and three were female with mean age of 25.3 years old (ranging from 18 to 44). All of the patients presented small AEDHs in regions corresponding to bleeding from branches of the MMA. Patients having traumatic lesions with mass effect, midline shift, or other associated intracranial injuries were not included in this study. Patients with moderate or large sized lesions underwent surgical evacuation and were not included as well. All patients had previous transient loss of consciousness following the trauma but had a score of 14 or 15 on the Glasgow Coma Scale on admission. Headache was the main complaint and no neurological deficits were observed.

The mean time from trauma to the patients' admission to our emergency center was 102 minutes (range: 25–260 minutes).  Table 1 provides a summary of the causes and other characteristics of the AEDHs.

Patients were treated according to ATLS protocol; patients with mild TBI underwent head CT scan. These patients with small epidural hematoma of middle fossa with temporal fracture were evaluated with noncontrast CT examination followed immediately by 3D CTA using a General Electric Light-Speed Advantage CT scanner with Advantage Windows 3D workstation (General Electric, Milwaukee, Wis). Noncontrast CT scans were performed with 5 mm contiguous axial sections through the posterior fossa followed by 10 mm contiguous axial sections to the vertex. CT angiography was performed using an injection of nonionic contrast at a rate of 3 mL/s for 80 mL initiated with a 15-second prescan delay. A 1 mm collimated helical scan with a 1 : 1 pitch was obtained from the cavernous carotid cephalic for at least 3.5 cm. On average, a total dose of 24 g of iodine was administered. Patients were kept under clinical observation in ICU. All patients underwent ipsilateral digital external carotid artery angiography which was performed within 12 hours of the initial study. The findings of angiotomography and digital angiography were reviewed by radiologists and angiographers, respectively, to ensure that the lesions were readily diagnosed without knowing the results of angiotomography. If any vascular lesion was found, embolization of the MMA and branches was performed after superselective injection with a microguidewire up to an area just before the arterial lesion had been reached. The ethics committee of our institution approved this study and informed consent for this study was obtained in all cases.

## 3. Results

The causes of head injury were traffic accidents (*n* = 6 patients), falls (*n* = 3 patients), and aggression (*n* = 2 patients). The largest hematoma had a thickness of 10 mm. Three of these patients presented traumatic MMA pseudoaneurysm. CT angiography was able to diagnose all of them, with dimensions ranging from 1.5 to 2.8 mm. Conventional angiography confirmed the findings of CT angiography, and the lesions presented with similar dimensions at both methods. No additional lesion was demonstrated by angiogram.

Scan duration was 35 to 42 seconds in all cases. Average CTA reconstruction time was 15 minutes. The acquisition time did not exceed 30 minutes including 3D reconstruction. The average period until discharge was 6.9 days (range: 5–9 days) for all patients and 2.7 days (range: 2–4 days) for the 3 patients with AEDHs treated with embolization. Embolization of the pseudoaneurysm itself or of the parent vessel was successfully performed in 3 patients. The postoperative course was uneventful and no complications related to the procedure were noted. All of the lesions were followed conservatively without surgical intervention and resolved within 21 days.

### 3.1. Illustrative Case

**Figure 1:**
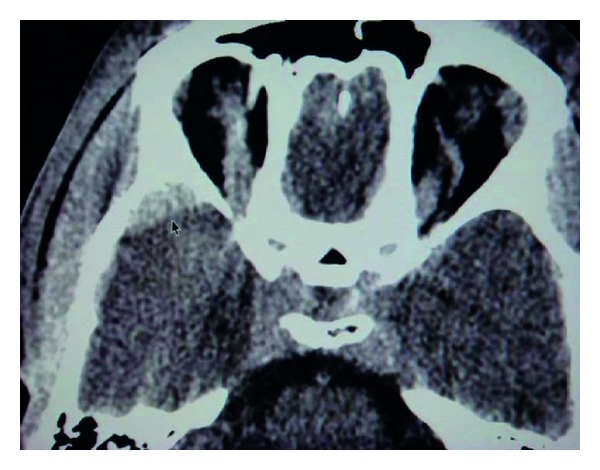
Computed tomography scan showing a small acute EDH in the right temporal region adjacent to the fracture.

**Figure 2:**
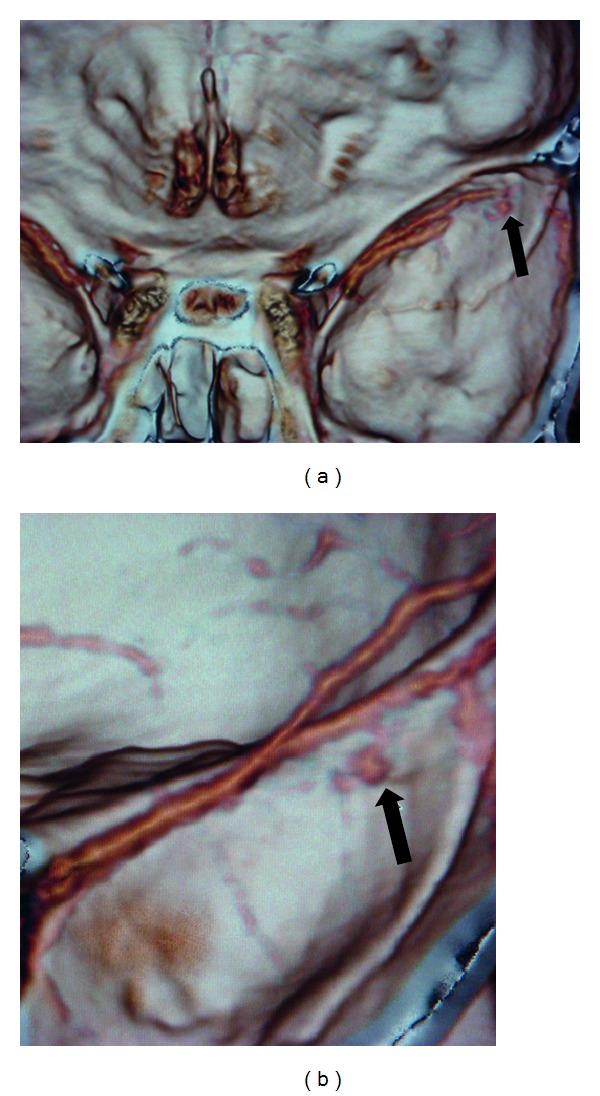
(a) Computed tomography angiography that displayed middle meningeal artery pseudoaneurysm. (b) More detailed image of the vascular lesion in computed tomography angiography with three-dimensional reconstruction of vessel and middle cranial fossa.

**Figure 3:**
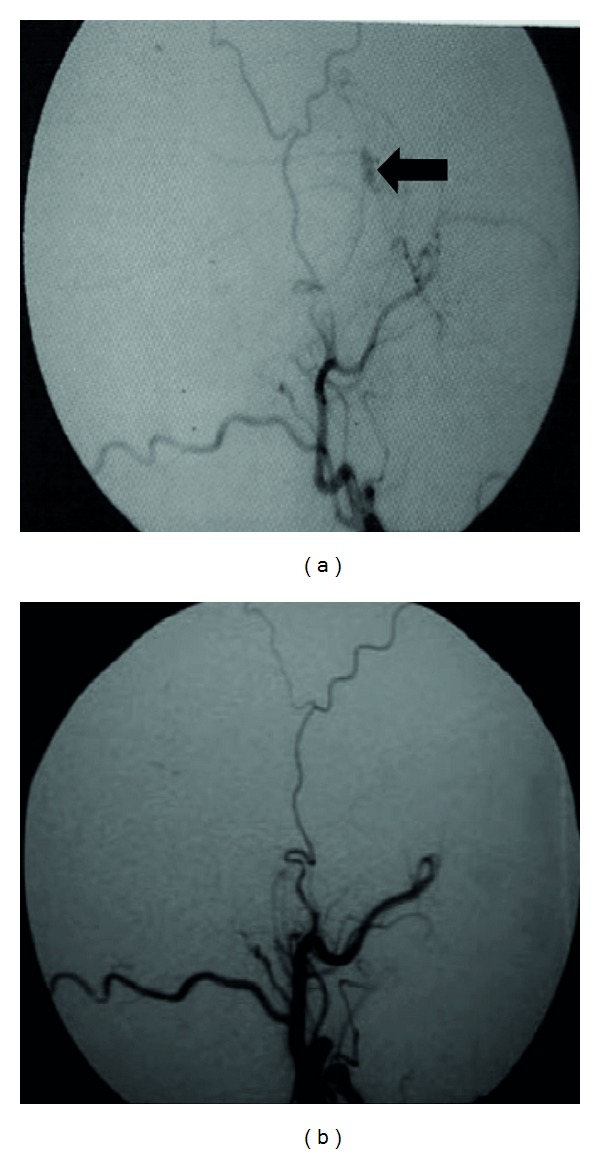
(a) Superselective external carotid artery angiography showing MMA pseudoaneurysm (arrow) before embolization. (b) External carotid artery angiography after pseudoaneurysm treatment showing complete embolization.

A 30-year-old man was admitted after a road traffic accident with transient loss of consciousness (20 minutes). In the emergency room, patient was conscious scoring 15 points in Glasgow Coma Scale. A neurological examination disclosed no abnormalities. A CT scan revealed a temporal linear fracture and a small AEDH in the right temporal region adjacent to the fracture (Figure 1). According to our protocol the patient underwent multislice angiotomography that displayed a pseudoaneurysm of MMA (Figures 2(a) and 2(b)). The patient remained in neurological observation. Superselective external carotid artery angiography confirmed MMA pseudoaneurysm diagnosis (Figures 3(a) and 3(b)) with similar characteristics with CTA findings. Embolization was performed uneventfully. Follow-up CT scans obtained after treatment did not show any hematoma enlargement and patient was discharged with no neurological abnormalities.

## 4. Discussion

Emergent surgical intervention is always the strategy of choice for patients with large AEDH [10, 11]. Nonetheless, controversies remain in management of small hematomas. Some studies have reported a spontaneous resolution of AEDHs without surgical procedure. However, these lesions may further enlarge, posing several risks to the patient. Enlargement of small AEDHs is probably caused by rebleeding of the initial vascular lesion that had been previously tamponaded [7].

Knuckey et al. [12] demonstrated that 65% of small AEDHs underwent expansion in the first 24 hours after trauma. There was a significant increase (at least 25 mm in the hematoma thickness) in 51% of the patients. Meder et al. [13] found that nearly one-fourth of AEDHs enlarged within 24 hours.

CT criteria that have been suggested for nonoperative management of AEDHs include a volume of less than 30 mL, a thickness of less than 15 mm, and a midline shift of less than 5 mm in some series [14, 15]. The site of the AEDH has been considered as a basic factor that influences the clinical course in nearly every report [14, 16]. Many authors have concluded that the temporal and posterior fossa regions are unsuitable locations for conservative management of AEDHs [17, 18]. Controversy is even greater in cases of temporal hematomas. In 2008 our group presented a series of patients with small hematomas who underwent conventional angiography. The incidence of pseudoaneurysm was 29% and endovascular management was carried out [7]. However, angiography carries some risks and presents associated morbidity. Thus far, there is no report evaluating the role of CT angiography in the diagnosis of such lesions. This study investigated the role of CT angiography on the management of MMA traumatic aneurysms.

CT angiography is a well-described technique in which contrast-enhanced helical CT scans are used to create a computer-generated three-dimensional depiction of blood vessels. CT angiography can provide reasonably detailed 3D angiograms that can be rotated freely in space on a computer workstation for viewing vascular anatomy from any projection [19]. Moreover, it is relatively quick to obtain CT angiography, which is an important issue for patients needing rapid surgical intervention and prompt diagnosis. It appears to be less invasive and safer than digital subtraction angiography. This allows punctual and safe identification of patients who presented with high risk of epidural hematoma enlargement.

From our data, we conclude that as many as 30% of patients with AEDHs and fractures crossing the MMA may have pseudoaneurysms. This data is congruent with previous report. Few occurrences of traumatic pseudoaneurysms of the MMA have been reported thus far [20]. There seems to be an association between pseudoaneurysms and temporal fractures (92%) and pseudoaneurysms and AEDHs (61%) [2]. The natural history of traumatic aneurysms is not well known, but progressive growth of traumatic aneurysms has been demonstrated on repeated angiograms [21, 22]. It is thought that they develop after a small tear in the meningeal artery, which is sealed off by a clot, then recanalize, and form a false lumen. These pseudoaneurysms gradually enlarge and can rupture at any time [7, 23]. Therefore, considering the risk of a secondary rupture, we suggest that the treatment of traumatic pseudoaneurysms must always be carried out without any delay.

Enlargement of the small epidural hematomas with fractures in the temporal region occurs routinely in medical practice and for that reason these patients are kept in strict neurologic observation. Rupture of these traumatic pseudoaneurysms of MMA would cause acute epidural hematoma. Previous studies reported that the prognosis of the rupture of the traumatic pseudoaneurysms was poor, and the mortality rate was 20% or higher [3, 23].

CTA seems to be an effective and less invasive method that can be applied in the diagnosis of these lesions and allow an early management in patients with small hematoma. In our study we found vascular lesions in 3 of 11 patients with the radiological features and dimensions confirmed by angiography. All lesions were identified by CTA and no additional case was diagnosed only by conventional angiogram. Based on this fact, we recommend CTA as the primary diagnostic tool for traumatic MMA pseudoaneurysm and if confirmed, this lesion should be treated with embolization, allowing early hospital discharge.

## 5. Conclusions

This study confirms the results of previous report that estimates the incidence of posttraumatic MMA aneurysms in 30% [7] and demonstrates that CTA presents the same accuracy as conventional angiogram in the diagnosis of MMA pseudoaneurysms. These results suggest that CTA may replace safely and effectively conventional angiogram and should constitute the main diagnostic resource in this clinical scenario.

## Figures and Tables

**Table 1:** Summary of the patients, causes, and radiological characteristics.

Patient	Sex	Age	Trauma mechanism	Hematoma location	Angio-CT	Angiography	GCS	GOS
1	M	19	Traffic accident	Temporal	Pseudoaneurysm	Pseudoaneurysm	15	5
2	M	18	Aggression	Temporal	Normal	Normal	15	5
3	F	19	Traffic accident	Temporal	Normal	Normal	15	5
4	M	29	Traffic accident	Temporal	Pseudoaneurysm	Pseudoaneurysm	14	5
5	M	32	Fall	Temporal	Normal	Normal	15	5
6	M	27	Aggression	Temporal	Normal	Normal	14	5
7	M	25	Traffic accident	Temporal	Normal	Normal	15	5
8	F	39	Aggression	Temporoparietal	Normal	Normal	14	5
9	M	30	Traffic accident	Temporal	Pseudoaneurysm	Pseudoaneurysm	15	5
10	F	44	Fall	Temporoparietal	Normal	Normal	15	5
11	M	32	Traffic accident	Temporal	Normal	Normal	14	5

## References

[1] Baykaner K, Alp H, Ceviker N, Keskil S, Seckin Z (1988). Observation of 95 patients with extradural hematoma and review of the literature. *Surgical Neurology*.

[2] Bezircioglu H, Ersahin Y, Demircivi F, Yurt I, Donertas K, Tektas S (1996). Nonoperative treatment of acute extradural hematomas: analysis of 80 cases. *The Journal of Trauma*.

[3] Bruneau M, Gustin T, Zekhnini K, Gilliard C (2002). Traumatic false aneurysm of the middle meningeal artery causing an intracerebral hemorrhage: case report and literature review. *Surgical Neurology*.

[4] Bullock MR, Chesnut R, Ghajar J (2006). Surgical management of acute epidural hematomas. *Neurosurgery*.

[5] Chen TY, Wong CW, Chang CN (1993). The expectant treatment of “asymptomatic” supratentorial epidural hematomas. *Neurosurgery*.

[6] Cucciniello B, Martellotta N, Nigro D, Citro E (1993). Conservative management of extradural haematomas. *Acta Neurochirurgica*.

[7] de Andrade AF, Figueiredo EG, Caldas JG (2008). Intracranial vascular lesions associated with small epidural hematomas. *Neurosurgery*.

[8] Beer-Furlan A, de Almeida CC, Noleto G, Paiva W, Ferreira AA, Teixeira MJ (2013). Dural sinus and internal jugular vein thrombosis complicating a blunt head injury in a pediatric patient. *Child's Nervous System*.

[9] Ersahin Y, Mutluer S (1993). Posterior fossa extradural hematomas in children. *Pediatric Neurosurgery*.

[10] Jamous MA, Abdel Aziz H, Al Kaisy F, Eloqayli H, Azab M, Al-Jarrah M (2009). Conservative management of acute epidural hematoma in a pediatric age group. *Pediatric Neurosurgery*.

[11] Kim JH, Yim MB, Lee CY, Kim IM (2001). Surgical management of pseudoaneurysm. *Journal of Korean Neurosurgical Society*.

[12] Knuckey NW, Gelbard S, Epstein MH (1989). The management of ’asymptomatic’ epidural hematomas. A prospective study. *Journal of Neurosurgery*.

[13] Meder J-F, Gaston A, Merienne L, Godon-Hardy S, Fredy D (1992). Traumatic aneurysms of the internal and external carotid arteries. One case and a review of the literature. *Journal of Neuroradiology*.

[14] Okumura H, Tenjin H, Ueda S (1998). A case of traumatic pseudoaneurysm of the middle meningeal artery treated with endovascular surgery. *Neurological Surgery*.

[15] Paiva WS, de Andrade AF, Mathias Júnior L (2010). Management of supratentorial epidural hematoma in children: report on 49 patients. *Arquivos de Neuro-Psiquiatria*.

[16] Ross IB (2009). Embolization of the middle meningeal artery for the treatment of epidural hematoma. *Journal of Neurosurgery*.

[17] Sagher O, Ribas GC, Jane JA (1992). Nonoperative management of acute epidural hematoma diagnosed by CT: the neuroradiologist’s role. *American Journal of Neuroradiology*.

[18] Sakai H, Takagi H, Ohtaka H, Tanabe T, Ohwada T, Yada K (1988). Serial changes in acute extradural hematoma size and associated changes in level of consciousness and intracranial pressure. *Journal of Neurosurgery*.

[19] Schwartz RB, Tice HM, Hooten SM, Hsu L, Stieg PE (1994). Evaluation of cerebral aneurysms with helical CT: correlation with conventional angiography and MR angiography. *Radiology*.

[20] Servadei F, Vergoni G, Staffa G (1995). Extradural haematomas: how many deaths can be avoided? Protocol for early detection of haematoma in minor head injuries. *Acta Neurochirurgica*.

[21] Singh M, Ahmad FU, Mahapatra AK (2006). Traumatic middle meningeal artery aneurysm causing intracerebral hematoma: a case report and review of literature. *Surgical Neurology*.

[22] Sullivan TP, Jarvik JG, Cohen WA (1999). Follow-up of conservatively managed epidural hematomas: implications for timing of repeat CT. *American Journal of Neuroradiology*.

[23] Wu X, Jin Y, Zhang X (2014). Intraparenchymal hematoma caused by rupture of the traumatic pseudoaneurysm of middle meningeal artery. *Journal of Craniofacial Surgery*.

